# Case Report: Atypical anti-GBM nephritis coexisting with Henoch-Schönlein purpura nephritis: exploring the pathogenic nexus

**DOI:** 10.3389/fimmu.2025.1641090

**Published:** 2025-09-17

**Authors:** Yongxiu Huang, Caihong Liu, Wei Wei, Yanbin Lin, Sirong Tao, Yuliang Zhao

**Affiliations:** ^1^ Department of Nephrology, Kidney Research Institute, West China Hospital of Sichuan University, Chengdu, China; ^2^ Department of Nephrology, West China Xiamen Hospital of Sichuan University, Xiamen, Fujian, China; ^3^ Department of Pathology, West China Second Hospital, Sichuan University, Chengdu, China

**Keywords:** atypical anti-GBM nephritis, HSPN, renal biopsy, plasmapheresis, immunosuppressants

## Abstract

**Background:**

While anti-glomerular basement membrane (GBM) nephritis typically manifests with circulating antibodies targeting the GBM, atypical seronegative variants may occur. Henoch-Schönlein purpura nephritis (HSPN) is characterized by IgA-dominant immune complex deposition. The simultaneous presentation of these two distinct immune-mediated glomerulopathies poses unique diagnostic and therapeutic dilemmas, with limited cases reported in the literature.

**Case presentation:**

We describe a 16-year-old female presenting with rapidly progressive glomerulonephritis and cutaneous purpura. Initial serological testing was negative for anti-GBM antibodies. Renal biopsy was performed, with light microscopy showing segmental glomerulosclerosis. Immunofluorescence demonstrated distinctive dual deposition patterns: mesangial IgA consistent with HSPN and linear IgG along with capillary walls and GBM, confirming concurrent atypical anti-GBM nephritis. The patient responded favorably to combination therapy including glucocorticoids, immunosuppressants, and plasmapheresis, with subsequent improvement in renal function and resolution of symptoms.

**Conclusion:**

This case illustrates the diagnostic challenges posed by seronegative anti-GBM nephritis with HSPN overlap, emphasizing the critical role of histopathological examination in establishing the diagnosis. Our experience supports the efficacy of early, aggressive immunosuppressive therapy in such complex presentations. These findings warrant further investigation into the possible shared pathogenic mechanisms of these two disease entities.

## Background

Anti-glomerular basement membrane (GBM) disease is classically characterized by circulating antibodies targeting the GBM, leading to rapidly progressive glomerulonephritis (RPGN) often accompanied by pulmonary hemorrhage (Goodpasture syndrome) (1). However, atypical variants exist where patients present without pulmonary involvement and demonstrate negative or low-titer anti-GBM antibodies (2). These seronegative cases pose significant diagnostic challenges, particularly when coexisting with other glomerular diseases (3). Henoch-Schönlein purpura nephritis (HSPN), the renal manifestation of IgA vasculitis, typically presents with cutaneous purpura, arthritis, and abdominal pain, with renal involvement characterized by IgA-dominant immune complex deposition (4). The concurrent presentation of these two distinct immune-mediated glomerulopathies - one mediated by anti-GBM IgG antibodies and the other by IgA immune complexes - represents a rare clinical scenario that remains poorly understood (5). This combination suggests possible shared pathogenic mechanisms involving vascular endothelial injury and aberrant immune complex deposition (6).

## Case presentation

A 16-year-old female presented to our nephrology department with a 6-year history of recurrent bilateral lower limb purpura and a 5-month history of progressive edema. The patient’s medical history revealed that six years prior to admission, she developed persistent non-blanching skin rashes on both lower extremities of unknown etiology. She was diagnosed with HSPN at an outside institution and treated with oral prednisone, which was gradually tapered and discontinued after six months of therapy. Five months before the current admission, the patient developed new-onset lower extremity edema and was rehospitalized. Treatment at that time included prednisone and mycophenolate mofetil for immunosuppression. Two months prior to the current admission, the patient developed severe hypertension, with blood pressure readings as high as 177/119 mmHg.

Upon admission, physical examination revealed a blood pressure of 138/93 mmHg without active cutaneous rash or subcutaneous hemorrhage. Initial laboratory workup demonstrated anemia (hemoglobin 90 g/L), impaired renal function (urea 11.3 mmol/L, creatinine 278 μmol/L, uric acid 470 μmol/L, estimated GFR 20.93 ml/min/1.73 m²), and negative serological markers, including anti-GBM antibody, ANA, dsDNA, ENA, and ANCA, with no M protein detected on immunofixation or serum protein electrophoresis. Urinalysis showed significant proteinuria (3+ qualitative, 3.50 g/24h) with a protein/creatinine ratio of 0.312 g/mmol Cr and an albumin/creatinine ratio of 2281.7 mg/g, along with mild microscopic hematuria (5 RBCs/HPF). Renal ultrasound revealed that both kidneys exhibited normal size and shape, but there was increased echogenicity of the renal parenchyma with loss of corticomedullary differentiation. The results suggested that the bilateral renal parenchyma was damaged, and the kidney size was 10.9×5.0×5.2 cm (right) and 10.6×4.9×4.7 cm (left).

**Figure 1:**
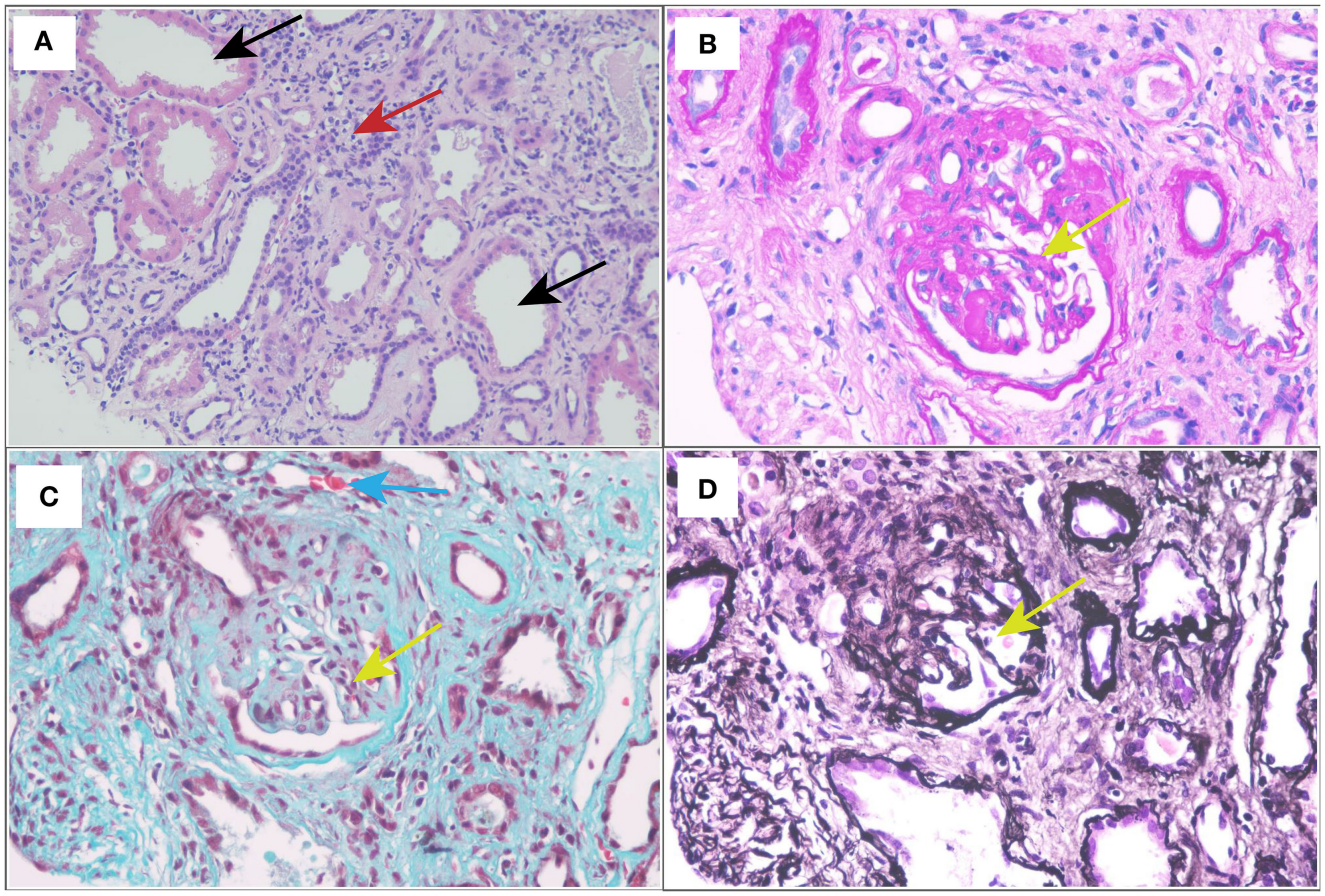
Light and electron micrographs: **(A)** HE stain (×400). **(B)** Periodic acid-Schiff (PAS) stain (×400). **(C)** Masson staining (×400). **(D)** Periodic acid-silver methenamine (PASM) (×400). Glomerular sclerosis (yellow arrow); dilated renal tubules (black arrows); renal interstitial fibrosis with lymphocytes, monocytes, and neutrophils infiltration (red arrow); the small artery wall was slightly thickened (blue arrow).

**Figure 2:**
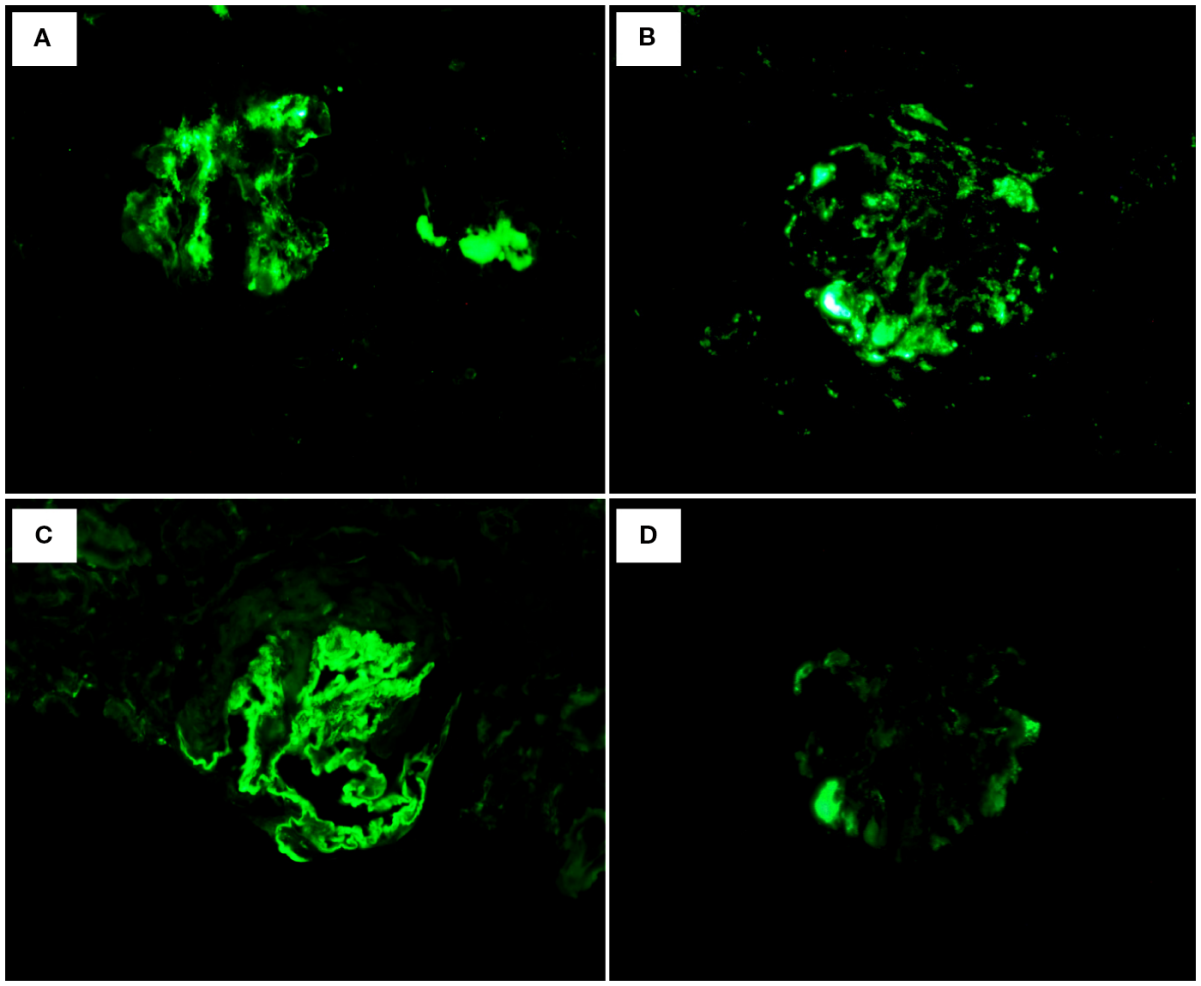
Immunofluorescence: **(A)** IgA: mesangial (++). **(B)** C3: mesangial (++). **(C)** IgG: Linear deposition of glomerular vascular wall (+++). **(D)** IgM: (+-).

**Figure 3:**
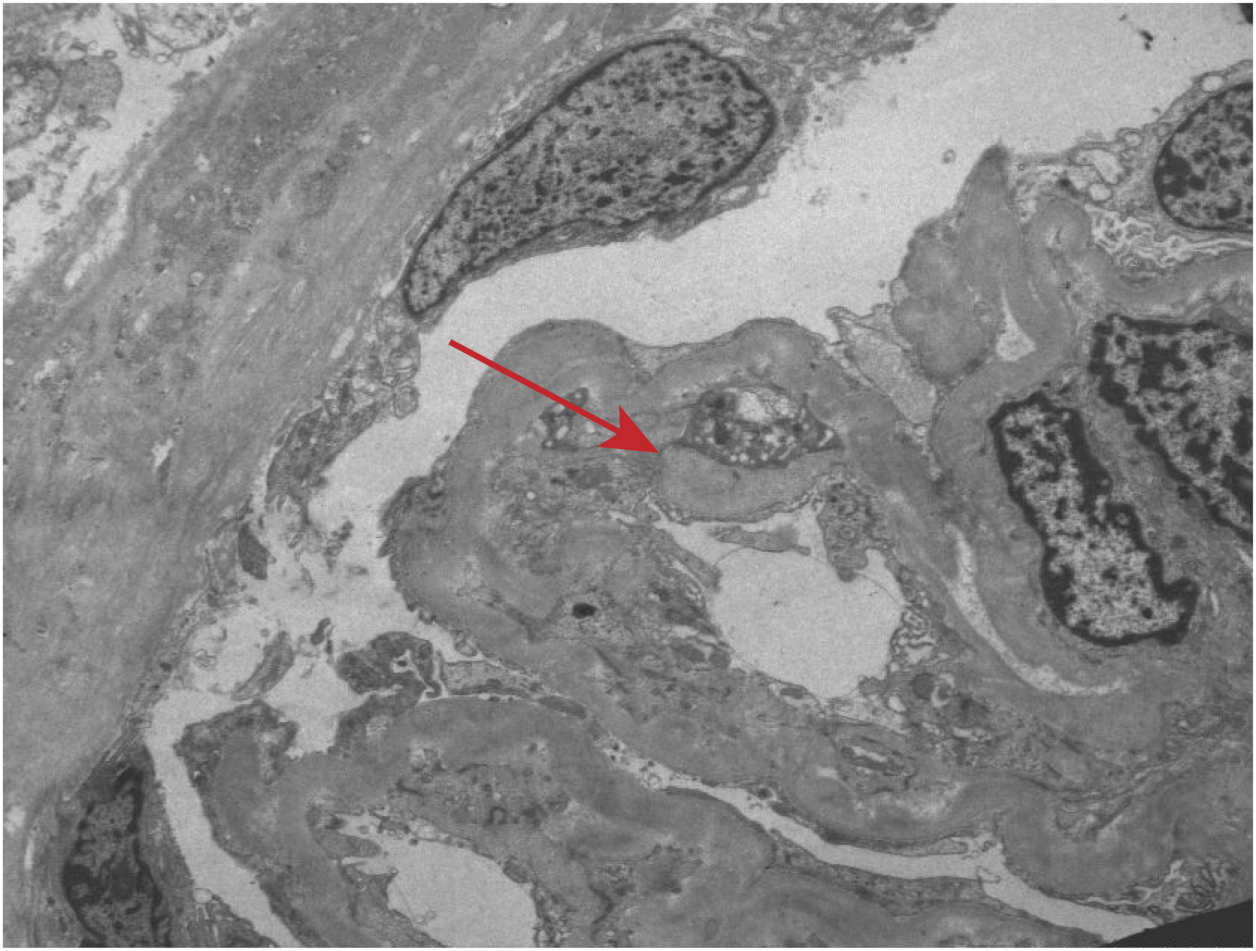
Electron microscopic observation. Irregular thickening and rupture of GBM, ischemic glomerular changes accompanied by mesangial proliferation and extensive foot process fusion (red arrow).

Renal biopsy was performed two days after admission, revealing significant pathological changes across all microscopic modalities. Light microscopy demonstrated advanced glomerular damage with 10 out of 12 glomeruli showing global sclerosis, while the remaining two exhibited segmental sclerosis and adhesion of Bowman’s capsule. The GBM displayed spherical vacuolar degeneration with shrinkage, accompanied by partial capillary lumen occlusion. Tubulointerstitial involvement was prominent, featuring approximately 60% tubular atrophy with moderate to severe epithelial cell degeneration, focal tubular dilatation, and epithelial cell shedding. The interstitium showed matching 60% fibrosis with lymphocytic, monocytic, and neutrophilic infiltration (**Figure 1**). Immunofluorescence studies of paraffin-embedded tissue revealed dual deposition patterns: mesangial IgA (++) and complement C3 (++), consistent with HSPN, along with linear IgG (+++) deposition along glomerular capillary walls, suggestive of anti-GBM nephritis (**Figure 2**). Electron microscopy further confirmed irregular thickening and rupture of GBM, ischemic glomerular changes accompanied by mesangial proliferation and extensive foot process fusion (**Figure 3**). The comprehensive histopathological evaluation supported a final diagnosis of sclerosing glomerulonephritis, with the collective findings most compatible with HSPN and concurrent anti-GBM antibody-mediated renal damage, particularly given the characteristic linear IgG deposition pattern.

**Figure 4:**
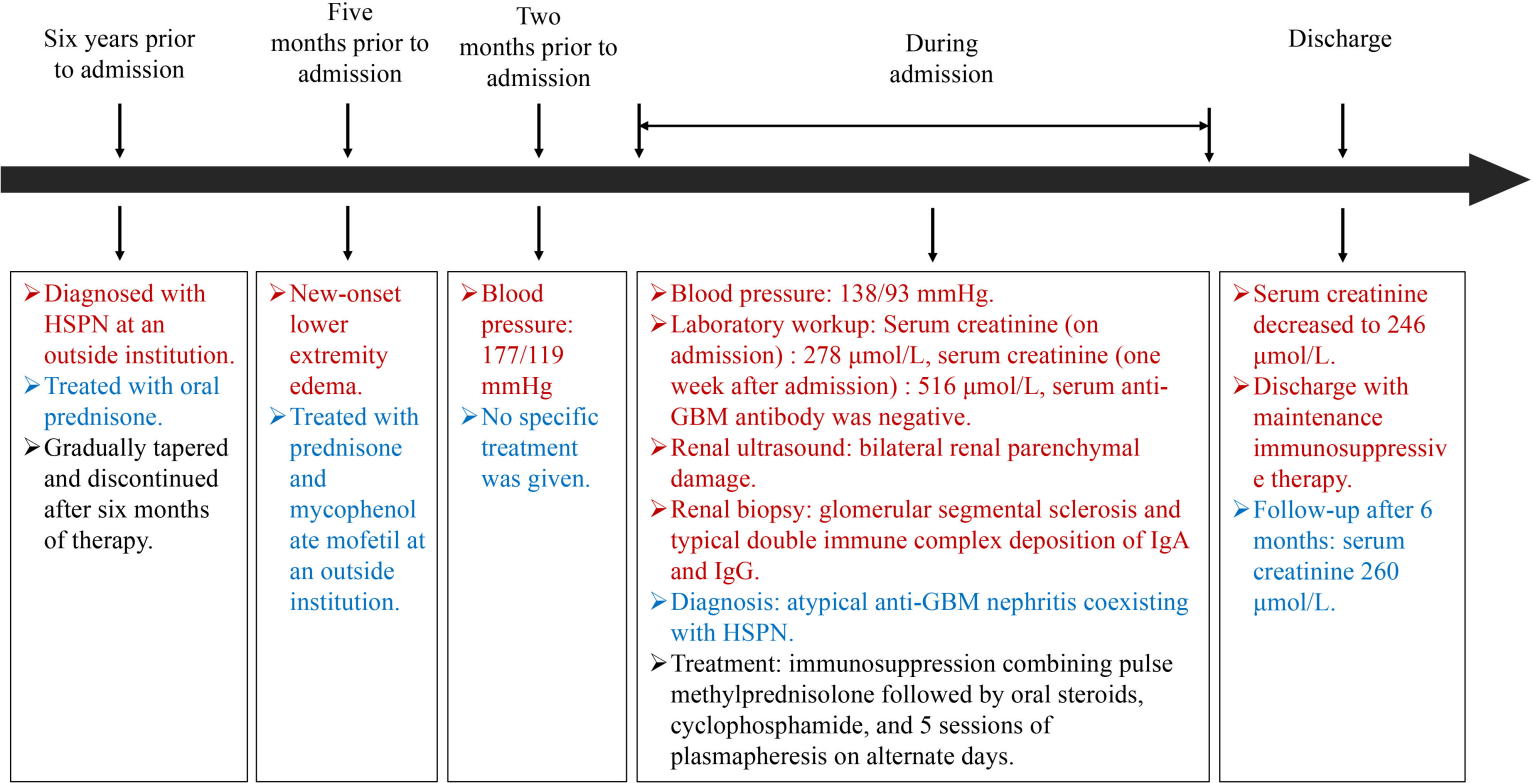
Timeline. Clinical history and therapeutic interventions of the case.

Following admission, the patient was initiated on comprehensive supportive therapy including antihypertensive treatment, anemia management, and prophylactic antibiotics. However, subsequent laboratory tests demonstrated progressive clinical deterioration with serum creatinine rising to 516 μmol/L, urea 16.7 mmol/L, uric acid 529 μmol/L, and eGFR declining to 9.91 mL/min/1.73 m², accompanied by hypoalbuminemia (33.6 g/L). In light of this rapidly worsening renal function and the biopsy findings suggestive of atypical anti-GBM nephritis, an aggressive therapeutic regimen was implemented: immunosuppression combining pulse methylprednisolone (0.8g*3d) followed by oral steroids, cyclophosphamide (0.2g q.o.d), and 5 sessions of double-filtration plasmapheresis on alternate days (7). This aggressive approach yielded significant improvement with serum creatinine decreasing to 246 μmol/L, allowing for discharge with maintenance immunosuppressive therapy. The patient’s renal function remained stable (serum creatinine 260 μmol/L) according to the most recent follow-up after 6 months (**Figure 4**).

## Discussion

Atypical anti-GBM nephritis coexisting with HSPN represents a rare and diagnostically challenging clinical entity (5). HSPN typically manifests with the classic triad of cutaneous purpura, arthralgia, and renal impairment (4), characterized pathologically by IgA-dominant immune complex deposition in small vessel walls (2) affecting multiple organ systems, including the skin, joints, gastrointestinal tract, and kidneys (3). In contrast to classic anti-GBM disease, the atypical variant demonstrates distinct clinicopathological features (6). Clinically, atypical anti-GBM nephritis presents with milder manifestations (8), notably lacking the pulmonary hemorrhage (9). Instead, patients predominantly exhibit urinary abnormalities (hematuria and proteinuria) with varying degrees of renal dysfunction. In this case, the patient’s urine analysis suggested mild hematuria. Notably, a single urine analysis may have inherent limitations in terms of repeatability and precision, while fluctuations in hematuria are common along with the course of the disease (10). Secondly, histological examination suggested a subacute lesion process. When the urine analysis results were obtained, the peak period of acute inflammatory activity may have subsided, and usually, obvious hematuria occurred during the acute inflammatory activity period (11). In addition, the patient presented with HSPN and anti-GBM antibody-negative overlap disease (12), which is a rare atypical combination that may lead to a unique clinical phenotype and is worthy of further observation and analysis to better describe its clinical process.

Serologically, atypical cases frequently show negative or low-titer circulating anti-GBM antibodies, contrasting sharply with the high antibody titers typically observed in classical anti-GBM disease (13). This serological discrepancy, combined with the coexistence of IgA deposition, creates significant diagnostic complexity requiring comprehensive histological evaluation in this case (14). The seronegativity observed in atypical anti-GBM disease may be attributed to several distinct pathophysiological mechanisms. First, the immunological profile differs fundamentally from classic anti-GBM nephritis. While typical cases involve direct antibody targeting of the α3 chain of type IV collagen in the GBM, forming characteristic linear immune deposits (13), atypical variants may involve: (1) antibodies against alternative basement membrane components (e.g., tubular basement membrane antigens), (2) non-antibody mediated immune mechanisms (immune complex deposition or T-cell mediated injury) (15), or (3) below-threshold antibody titers that fall beneath the detection limits of conventional assays (16). Second, temporal dynamics of antibody production may contribute, as some patients may experience antibody level fluctuations - initial antibody presence may wane naturally or in response to therapy, potentially yielding negative results during testing windows despite persistent clinical manifestations (17). Third, methodological limitations of current diagnostic techniques (ELISA and immunofluorescence assays) may fail to detect certain antibody subtypes due to restricted epitope recognition or insufficient sensitivity (18, 19). Finally, concurrent autoimmune conditions (e.g., systemic lupus erythematosus) or immune complex-mediated nephritides (e.g., HSPN in this case) may generate competing immunological signals that either mask true anti-GBM antibodies or redirect the immune response toward alternative pathways (5). This immunological complexity underscores the necessity of combining serological testing with comprehensive histological evaluation in suspected atypical cases. As in this case, it may be that the patient’s autoantibodies are non-classical epitopes that cannot be recognized, such as laminin-521 and perlecan (20). It may also be that local antibodies are produced in the kidney, with minimal systemic exposure (21). Linear immunoglobulin staining and significant complement deposition in the patient’s renal biopsy confirmed the antibody-mediated process, which proved that renal biopsy was still the gold standard for the etiological diagnosis of RPGN (22).

The pathological features of atypical anti-GBM nephritis demonstrate distinct characteristics across microscopic examinations. Light microscopy typically reveals capillary proliferative glomerulonephritis, membranoproliferative glomerulonephritis, mesangial proliferative glomerulonephritis, or focal segmental glomerulosclerosis, with occasional focal crescent formation and fibrinoid necrosis, contrasting with the diffuse crescent formation and fibrinoid necrosis seen in typical cases (23). Immunofluorescence microscopy shows linear immunoglobulin deposition along the GBM, predominantly of IgG1 and IgG4 subtypes, with approximately one-third of cases exhibiting concurrent IgG2 deposition and notably less frequent IgG3 deposition - a pattern potentially attributable to the strong complement-binding capacity of IgG3 compared to the weaker complement-binding abilities of IgG4 and IgG2 (24). This manifests as predominantly single IgG subtype deposition in atypical cases, in contrast to the polymorphic antibody deposition characteristic of typical anti-GBM disease (23). Electron microscopy findings in atypical anti-GBM disease mirror those of typical cases in demonstrating no electron-dense deposits along the GBM, though some patients may exhibit sparse electron-dense deposits in the mesangial area, subendothelial space, or subepithelial region (25, 26).

The coexistence of atypical anti-GBM nephritis with HSPN involves dual pathological mechanisms of glomerular injury and purpura-induced vascular inflammation (2), where their synergistic interaction accelerates renal microvascular destruction and local inflammation, leading to rapid progression of renal damage (4). Differential diagnosis requires careful distinction from several clinically similar conditions, including Goodpasture syndrome (13), systemic lupus erythematosus, and IgA nephropathy. While typical anti-GBM nephritis characteristically presents with pulmonary-renal syndrome featuring acute renal failure and pulmonary hemorrhage, the atypical variant typically manifests with isolated renal symptoms (27). Lupus nephritis may mimic these renal manifestations but typically occurs in the context of systemic involvement such as malar rash, arthritis, or other constitutional symptoms (28). IgA nephropathy, though sharing clinical features like proteinuria and hematuria (29), demonstrates distinct pathological characteristics with predominant mesangial IgA deposition on immunofluorescence staining (14), contrasting with the linear IgG pattern of anti-GBM nephritis and featuring cutaneous purpura in HSPN (16).

The management of atypical anti-GBM nephritis requires a tailored approach based on disease severity, progression, and patient comorbidities, typically involving combined immunosuppressive and supportive therapies (17). Immunosuppressive treatment focuses on attenuating hyperactive immune responses and mitigating antibody-mediated GBM injury (19), utilizing high-dose corticosteroids, cytotoxic agents (cyclophosphamide), and plasmapheresis/immunoadsorption (18, 21, 30). In this case, the patient’s serum creatinine level rose sharply from 278 μmol/L to 516 μmol/L in a short period of time. This rapid decline in renal function constitutes a medical emergency and requires immediate intervention to stop sustained renal injury (31). Although the renal biopsy did not reveal active crescents—potentially due to sampling limitations—immunofluorescence demonstrated linear immunoglobulin deposition. It’s possible that unsampled glomeruli may harbor active and treatable lesions, such as cellular crescents or necrotizing inflammation. According to the KDIGO 2021 Clinical Practice Guidelines for Glomerular Disease Management, for suspected anti-GBM patients with clinical manifestations and/or pathological clues who are not dialysis-dependent, empirically combined immunosuppression and plasmapheresis is indicated (31). After plasmapheresis and immunosuppression, the patient’s renal function showed a significant improvement, indicating that there is indeed a positive, therapeutic response to the immune regulation process (11). Besides, comprehensive supportive care remains equally critical, particularly during acute phases with significant renal impairment (27), addressing common complications including edema, hypertension, and electrolyte imbalances through vigilant monitoring of the inner environment (32).

After treatment, the patient’s renal function was partially restored, although the renal biopsy showed late chronic changes (10/12 of the sclerotic glomeruli, 60% of interstitial fibrosis), which may be due to the inherent sampling variability of the renal biopsy. When a very small part of the kidney is sampled, it is usually 10–20 of the nearly one million glomeruli. The sampled cortex contains a disproportionately high scar area, and other areas of the kidney may contain a higher proportion of glomeruli. These glomeruli have active and potentially reversible damage (such as cell crescents or acute renal tubular injury), responding to active immunosuppression and plasmapheresis. In addition, the clinical course of the patient was an “acute-on-chronic” disease phenotype. Serum creatinine rose sharply from an elevated baseline level, indicating that a new, violent inflammation was superimposed on the background of pre-existing chronic kidney disease, and our treatment targets this acute part. The subsequent decrease in creatinine returned to the elevated baseline, representing a recovery of residual viable nephron function.

## Conclusion

This case report describes a rare presentation of atypical anti-GBM nephritis coexisting with HSPN, characterized by serum anti-GBM antibody negativity. The diagnosis was confirmed by renal biopsy findings demonstrating double immune complex deposition of IgA and IgG. Our experience supports the therapeutic efficacy of a multimodal approach combining glucocorticoids, immunosuppressive agents, and plasmapheresis. However, the clinical course and pathophysiology of atypical anti-GBM nephritis with HSPN remain under investigated. These observations underscore the need for further research to elucidate disease mechanisms and optimize treatment strategies for suspected anti-GBM nephritis patients with seronegativity.

## Data Availability

The raw data supporting the conclusions of this article will be made available by the authors, without undue reservation.
